# Seeing the forest for the genes: using metagenomics to infer the aggregated traits of microbial communities

**DOI:** 10.3389/fmicb.2014.00614

**Published:** 2014-11-12

**Authors:** Noah Fierer, Albert Barberán, Daniel C. Laughlin

**Affiliations:** ^1^Cooperative Institute for Research in Environmental Sciences, University of ColoradoBoulder, CO, USA; ^2^Department of Ecology and Evolutionary Biology, University of ColoradoBoulder, CO, USA; ^3^Environmental Research Institute, School of Science, University of WaikatoHamilton, New Zealand

**Keywords:** metagenomics, traits, community-aggregated traits, microbial diversity, microbial ecology

## Abstract

Most environments harbor large numbers of microbial taxa with ecologies that remain poorly described and characterizing the functional capabilities of whole communities remains a key challenge in microbial ecology. Shotgun metagenomic analyses are increasingly recognized as a powerful tool to understand community-level attributes. However, much of this data is under-utilized due, in part, to a lack of conceptual strategies for linking the metagenomic data to the most relevant community-level characteristics. Microbial ecologists could benefit by borrowing the concept of community-aggregated traits (CATs) from plant ecologists to glean more insight from the ever-increasing amount of metagenomic data being generated. CATs can be used to quantify the mean and variance of functional traits found in a given community. A CAT-based strategy will often yield far more useful information for predicting the functional attributes of diverse microbial communities and changes in those attributes than the more commonly used analytical strategies. A more careful consideration of what CATs to measure and how they can be quantified from metagenomic data, will help build a more integrated understanding of complex microbial communities.

## WHY MICROBIAL ECOLOGISTS ARE STILL LOST IN ‘TERRA INCOGNITA’

Ever since van Leeuwenhoek first peered through a microscope, it has been recognized that most environments harbor diverse and complex microbial communities. Whether we are studying soil, the human gut, marine sediments, or lake waters, we find many taxa that have unknown, or at least poorly described, ecological characteristics. Our descriptions of microbial communities are littered with question marks just as maps of continents were once littered with labels of ‘*terra incognita*’ by European explorers.

Recent methodological advances, most prominently advances in DNA sequencing, have provided unique insight into the structure and function of complex microbial communities, thereby improving our ability to chart ‘*terra incognita.*’ This is particularly true for those environments, like soils and sediments, which harbor many taxa that are resistant to laboratory isolation (Whitman et al., 1998; Fierer and Lennon, 2011). It is now routine for researchers to use shotgun metagenomics, randomly sequencing from a pool of whole-community DNA extracted from environmental samples (Handelsman, 2004), to characterize complex microbial communities and their functional capabilities. Shotgun metagenomics is by no means the only approach available – but, fueled by rapid declines in sequencing costs, shotgun metagenomes are an increasingly available source of data that can be mined to characterize microbial communities.

One of the explicit goals (and promises) of many shotgun metagenomic projects is to understand the broader functional and ecological characteristics of microbial communities, insight that cannot necessarily be gleaned from the sequencing of taxonomic or phylogenetic marker genes. These arguments are convincing because there is already evidence that such approaches can be used to better understand the function of complex microbial communities, whether those functional capabilities are related to phosphate removal from wastewater (Martin et al., 2006), carbon cycling in permafrost soils (Mackelprang et al., 2011), or arsenic geochemistry in marine sediments (Plewniak et al., 2013). While these and many other studies highlight the utility of shotgun metagenomic analyses, it is often difficult to distinguish tangible advances from the hype.

We can use metagenomic data to develop testable predictions regarding the ecological attributes of microbial communities, but the approach is no panacea and inferring the functional capabilities of communities from metagenomic data remains difficult. One reason for this is that community-level attributes are the emergent properties of a diverse array of organisms interacting directly and indirectly in a myriad of ways under environmental conditions that are rarely static. Even if we could predict the ecological attributes of all individual taxa living in a given community (a Sisyphean task in most microbial habitats), the overall functional capabilities of that community and how it responds to changes in biotic or abiotic conditions, will remain difficult to predict. If we want to know how rapidly soil communities will decompose soil organic matter or how efficiently a gut microbial community will ferment ingested polysaccharides, it is insufficient to document the genes associated with the metabolism of various organic carbon pools and their relative abundances. Even in simple communities composed of well-described microbial taxa, predicting community-level metabolic properties from genomic or transcriptomic data can be challenging (Sieuwerts et al., 2010).

If the goal is to link microbial communities to processes, a critical step is to understand the ecological attributes of whole communities, not just the attributes of individual community members as the whole is rarely the sum of its parts. Here we argue that we could often do a better job of predicting the functional capabilities of whole communities by using the concept of community-aggregated traits (CATs) to glean more useful information from the terabases of shotgun metagenomic data being generated.

## WHAT ARE COMMUNITY-AGGREGATED TRAITS AND WHY ARE THEY USEFUL?

Functional traits can be measured at various levels of organization – from the level of individual cells, to species, to whole communities. For example, one could determine the size of individual bacterial cells in a water sample, the mean cell sizes for different bacterial species found in that sample, or the mean size of cells found in the whole sample. Community-level traits can be quantified either as a “community-weighted mean trait,” where the mean trait values for all taxa in a community are weighted by their relative abundances, or as a “CAT,” where the traits are measured from a random sample of individuals irrespective of their taxonomic identities.

We can calculate community-weighted mean traits from shotgun metagenomic data by reconstructing genomes, or parts of genomes, and using this genomic information to predict the characteristics of individual community members. This ‘bottom up’ approach has already been demonstrated to be useful for describing the putative functions of undescribed microbial taxa (e.g., Walsh et al., 2009; Hug et al., 2013) or for documenting taxa likely responsible for specific microbial processes (e.g., Howard et al., 2008). Likewise, we can calculate community-weighted mean traits from *a priori* information on the traits of specific microbial taxa (or lineages). While this approach is certainly appealing (e.g., Langille et al., 2013), it has some clear disadvantages if the traits of many microbial taxa, even closely related taxa, are not already known, as would be the case in many habitat types. Likewise, for those traits that are capable of being transferred across distantly related taxa via horizontal gene transfer (e.g., antibiotic resistance; Forsberg et al., 2012), trying to determine community-levels trait solely from taxonomic or phylogenetic information would be problematic.

An alternative approach is to calculate CATs directly from a community of interest without collecting any information on the identities of the taxa found in a given plot. For example, leaf traits in a plant community can be determined by remote sensing of canopy spectra (Homolová et al., 2013) or through random taxon-free sampling (Gaucherand and Lavorel, 2007) without having to measure trait averages for each plant species. In a similar manner, we can calculate CATs from shotgun metagenomic data as long as we assume that our metagenomes represent a random sampling of all microbial genomes present in that sample and that the traits of interest can actually be inferred from the genomic information. Such a ‘top–down’ approach has been widely used in microbial environments ranging from soil (Fierer et al., 2012) to marine waters (Ganesh et al., 2014) to the human gut (Greenblum et al., 2012). In all of these cases, much of the insight into community attributes came not from assigning genes to taxa, but rather by determining the relative abundances or presence/absence of genes and gene categories at the community-level of inquiry, not at the level of individual taxa.

CATs are often useful predictors of community-level properties because, according to the mass ratio hypothesis, species controls on community-level processes are in proportion to their relative abundances (Grime, 1998). The functional traits of abundant taxa will have more important influences on the functional properties of a community than the traits of subordinate taxa. There is strong empirical support for the mass ratio hypothesis from those studies that have explicitly tested the hypothesis in plant communities (reviewed in Lavorel and Grigulis, 2012). For example, Mokany et al. (2008) demonstrated that mean trait values were a good predictor of multiple ecosystem properties in grasslands, including litter decomposition rates and aboveground net primary productivity. Likewise, other studies have demonstrated the utility of using plant CATs to predict a wide range of processes and ecosystem properties from nitrification rates (Laughlin, 2011) to soil carbon dynamics (Garnier et al., 2004), and green biomass production (Lavorel et al., 2011).

**FIGURE 1:**
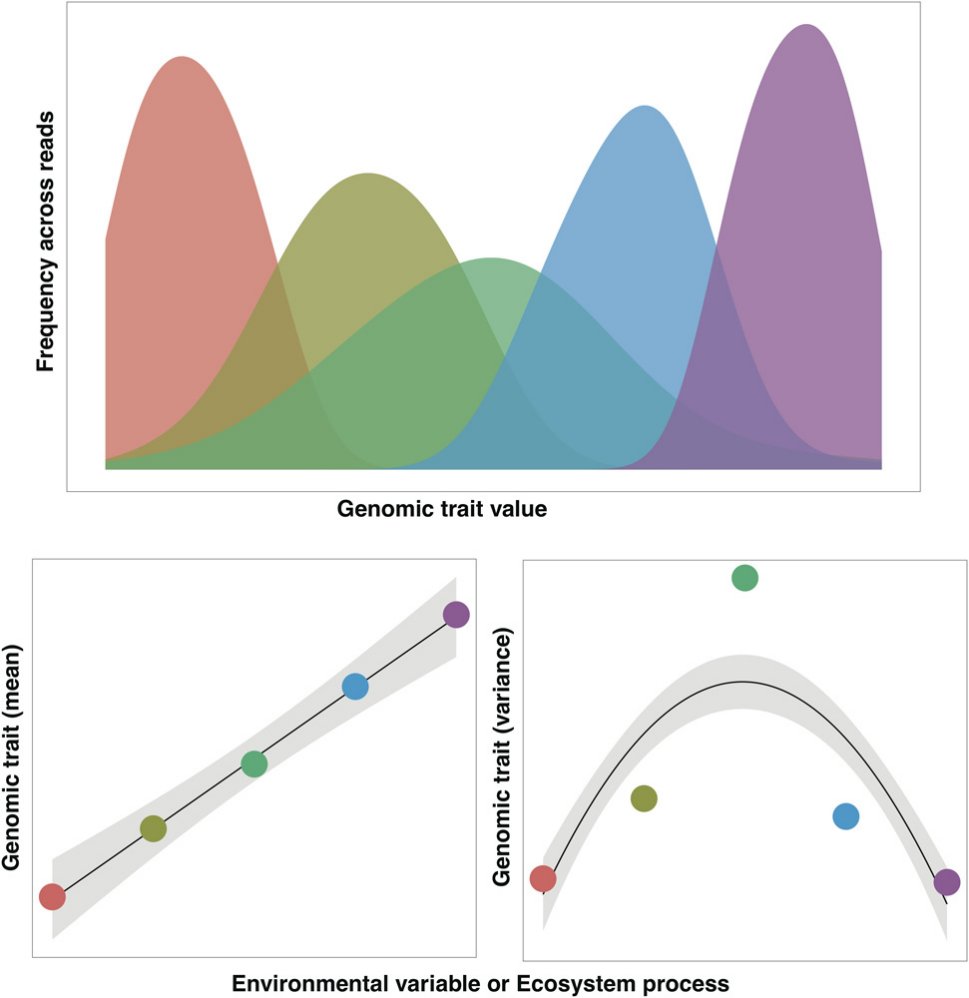
**Conceptual distribution of a community-aggregated trait (CAT) as inferred from the frequency of sequence reads from five different metagenomes (represented as different colors).** For an illustrative purpose, we can assume that the mean value of the trait increases along an environmental gradient or correlates with an ecosystem process of interest. Likewise, we can assume that the variance of this same trait is reduced at the extreme values of the gradient/process as a result of selective pressures. Trait distributions as conceptualized in this figure have been observed, for example, in the distribution of GC content across aquatic metagenomes (Barberan et al., 2012).

In many cases, the mass ratio hypothesis should also apply to microbial communities. For example, we might expect that the efficiency by which microbes mineralize nitrogen in a composting bio-reactor would be a function of the community mean for those traits associated with nitrogen mineralization rates. The appeal of the mass ratio hypothesis to ecologists working with highly diverse microbial communities is that we do not necessarily need to know the traits of all taxa to predict a process of interest, rather we just need to know the traits of the more dominant taxa (Grime, 1998). Of course, not all community-level processes will be predictable from the mass ratio hypothesis. For example, relatively rare ‘keystone’ taxa can have a disproportionate influence on certain processes (Paine, 1995) and biotic interactions could invalidate the mass ratio hypothesis (Vile et al., 2006). Moreover, the diversity of traits (the range or variance in trait values found in a given community) could often be more relevant (Violle et al., 2012) than just the mean CAT value (**Figure 1**). For example, under fluctuating environmental conditions, like those found in wet soils exposed to wide swings in O_2_ levels, the distribution of traits associated with O_2_ tolerance could be more informative for predicting community responses over time than simply measuring a mean community-level O_2_ preference (Pett-Ridge and Firestone, 2005). In theory, we should be able to use shotgun metagenomic data to calculate both means and variances of traits (**Figure 1**), based on the distribution of the relevant genes or gene categories indicative of the trait of interest within the community.

CATs have already proven useful for predicting some microbial processes. For example, soil microbial ecologists have long considered bacterial:fungal ratios to be a useful metric for understanding soil carbon dynamics because bacteria and fungi are considered to have distinct carbon use efficiencies (Bailey et al., 2002), an assumption that has been called into question (Strickland and Rousk, 2010). Likewise, the elemental composition of planktonic cells can often be used to predict biogeochemical dynamics in aquatic systems (Finkel et al., 2009) just as C:N ratios of heterotrophic microbial communities appear to influence nitrate accumulation rates in a wide range of systems (Taylor and Townsend, 2010). Some models have incorporated community-level information on microbial traits or functional guilds to predict litter decomposition rates (Allison, 2012), soil carbon cycling (Wieder et al., 2013), and patterns in marine phytoplankton biomass (Dutkiewicz et al., 2009). Although these models did not determine CATs empirically, they do demonstrate that models which incorporate shifts in CATs (or equivalent) are useful for predicting how microbial processes, even complex microbial processes likely driven by hundreds of microbial taxa, can effectively be predicted across space or time.

## WHICH MICROBIAL CATs TO MEASURE?

If we assume that CATs provide a useful means to describe the functional attributes of communities, we then have to identify the traits that are relevant and worth quantifying. There is a long list of microbial traits that could be useful (see examples in **Table 1**) and the list of traits that will be most relevant will depend on the study system and the research question. Collapsing phenotypic diversity into groups with shared traits has been one approach to simplify this complexity. For example, microbial taxa have been divided into groups based on shared life history characteristics (Fierer et al., 2007; Ho et al., 2013), groups defined by the source of carbon or energy (lithotrophs, heterotrophs, autotrophs), or divided into groups based on specific functional capabilities (N_2_-fixers, photosynthesizers, methylotrophs). While pragmatic, these functional categories over-simplify trait-level variability and it is likely more appropriate to define microbial trait space as a set of continuous, quantifiable variables, where microbes, or microbial communities, sit within this multidimensional space.

**Table 1:** Selected examples of microbial traits and the genes or genomic characteristics that could be used to calculate community-aggregated trait (CAT) values from shotgun metagenomic data.

Microbial trait	Selected genes, gene categories, or genomic characteristics that could be used to infer the trait value	Reference
Maximum growth rate	rRNA operon number, codon usage bias in highly expressed genes, rRNA/tRNA position	Lauro et al. (2009), Vieira-Silva and Rocha (2010)
Dormancy	Sporulation proteins, toxin–antitoxin systems, resuscitation-promoting factors	Lennon and Jones (2011)
Osmoregulation	Trehalose and peptidoglycan production	Culligan et al. (2012)
Ability to catabolize recalcitrant organic compounds	Genome size, secondary metabolite transport/metabolism	Konstantinidis and Tiedje (2004), Fierer et al. (2012)
Stress resistance (general)	Sigma factor subunits of RNA polymerases (e.g., *σ*^B^)	Kazmierczak et al. (2003), Marles-Wright and Lewis (2007)
Cold tolerance	Cold shock proteins, trehalose synthesis proteins	Varin et al. (2012)
Motility	Chemoreceptor, flagellar genes	Girgis et al. (2007)
Oxidative stress tolerance	Catalase, peroxidase, and polyketide synthase genes	Qin et al. (2012), Zhang et al. (2013)
Nitrogen/phosphorus affinities	Genes for membrane-bound nutrient uptake/transporters	Hewson et al. (2009), Eloe et al. (2011)
Resistance to toxic metals	COGs associated with heavy metal eﬄux pumps	Hemme et al. (2010), Eloe et al. (2011)
Antibiotic resistance	Genes for eﬄux pumps, ribosomal protection, enzymatic inactivators	Hu et al. (2013), Forsberg et al. (2014)

*References include examples of studies where that trait was inferred from either genomic or metagenomic data.*

There are 100s of traits that could possibly be measured for any organism – but it is often not necessary to measure every one of these traits to place species into multidimensional trait space (Laughlin, 2014). The key is to identify and measure those traits that are most relevant to the system in question and select those traits that are independent and that most effectively discriminate between taxa or communities. Such an approach has proven useful in plant ecology where a handful of plant traits can be used to accurately place species into multidimensional trait space (Laughlin et al., 2010; deBello et al., 2013) as many other plant traits are often correlated with this subset of traits. There are clearly similar trade-offs in microbial traits (Gudelj et al., 2010). For example, there are well-established trade-offs between growth rate and yield (Pfeiffer et al., 2001), between stress tolerance and the ability to compete for substrates (Ferenci and Spira, 2007), and between cell size and nutrient uptake rates (Young, 2006). Although the list of possible traits is enormous, there are inescapable morphological, physiological, and genetic constraints that narrow the list of possible trait combinations associated with different life history strategies.

Selecting CATs to study can often be done *a priori*. For example, we can assume that traits that confer tolerance to changes in water activity will likely be important if we are trying to predict soil C dynamics in arid or semi-arid systems (Lennon et al., 2012; Evans and Wallenstein, 2014). Likewise, we could assume that traits associated with nutrient uptake will have important controls on phytoplankton growth in many freshwater systems (Edwards et al., 2013). Sometimes the traits that might be relevant to predicting the community-level function are unknown or unexpected because the process itself or specific controls on the process are not well understood. For example, it would be difficult to predict *a priori* which CATs would be most relevant to understanding controls on nitrification by archaea given that physiologies remain poorly understood (You et al., 2009). In these cases, one could determine which CATs are most relevant to a process of interest by first measuring the community-level process across space and/or time and then empirically determining what CAT, or set of CATs, appears to be correlated with the measured changes in the process. In this manner, one could generate specific, testable predictions about how changes in CATs relate to changes in community-level processes and test them experimentally.

## INFERRING COMMUNITY-AGGREGATED TRAITS FROM METAGENOMIC DATA

Many microbial traits could be inferred from genomic or metagenomic data (**Table 1**) and there are many other examples of traits that could be inferred directly from either the presence/absence of specific genes, including specific metabolic capabilities (e.g., the ability to fix CO_2_ or oxidize elemental sulfur) or tolerances to specific environmental stressors (e.g., resistance to antibiotics or toxic metals). Other traits could be determined from genomic data but, in many cases, there will not be a single gene, or set of genes, whose presence or absence is directly associated with the trait. Instead, one would have to identify what gene abundances or gene ratios correlate with the trait of interest. For example, maximum growth rate is clearly an important trait, but there is no single gene that directly determines growth rate. However, a variety of genomic features (including number of rRNA copies, number of outer membrane proteins, presence of motility genes) have been shown to distinguish copiotrophic and oligotrophic taxa (Klappenbach et al., 2000; Lauro et al., 2009) with work by Vieira-Silva and Rocha (2010) demonstrating how such information could be used with metagenomic data to estimate microbial generation times in environmental samples. In many cases, the process of inferring specific CATs from metagenomic data will require analyzing genomes from individual taxa with well-characterized phenotypic traits or by analyzing metagenomes from communities across well-characterized gradients in CAT-space (Barberan et al., 2012). For example, there are likely genes, or combinations of genes, that could provide insight into the nutrient stoichiometries of individual taxa or whole communities, but these genes will have to be identified and their utility validated.

There are numerous caveats to consider when trying to use metagenomic data to quantify microbial CATs and nearly all of these caveats are shared by any study using metagenomes to infer the functional attributes of communities. First and foremost, traits are phenotypic characteristics of organisms and changes in metagenomes will not necessarily equate with changes in CATs. Metagenomes allow us to make some guesses and hypotheses about changes in CATs, but these hypotheses would need to be confirmed independently. This is particularly true for traits, like cell size (Young, 2006) and nutrient stoichiometries (Scott et al., 2012), which can exhibit a high degree of plasticity depending on environmental conditions. A related concern is that CAT estimates from metagenomes will reflect both active and inactive members of the community and in many environments, like soil, a large portion of the DNA pool isolated at a given point in time could come from cells that are dormant, inactive, or even lysed (Lennon and Jones, 2011). Perhaps a larger concern is that we are often going to be restricted to estimating CATs from that portion of the metagenome that has been annotated. In environmental metagenomes, typically >50% of the genes found in metagenomes are of undetermined function (Ellrott et al., 2010) and it is a big leap of faith to assume that those annotations are thorough and accurate (Schnoes et al., 2009). Although some traits can be inferred from unannotated metagenomic data (e.g., codon usage, Roller et al., 2013), the requirement that genes are first annotated is an important limitation in most environmental samples as the large fraction of genes that fall into the ‘known unknown’ category may obscure relationships between CATs and the community-level attributes one is trying to predict.

## CONCLUSION

Microbial ecologists are inherently interested in the details and complexities of microbial communities. This is not true for most people outside the field – they want to know what microbial communities do, how they can be managed, and how they impact our health and environmental quality. Thus, there is a need for quantifiable information on community-level functions and the relevant community-level properties. Unfortunately, the traits of most microbial taxa living in the environment remain poorly understood and trying to understand community-level properties from trait-based information on individual cells or individual taxa is often difficult, if not impossible. There is no method waiting on the horizon that will serve as a panacea to close these knowledge gaps. However, CATs represent a valuable conceptual approach that could be used by microbial ecologists to make better use of shotgun metagenomic data for predicting the functional capabilities of complex communities. The key is to determine what CATs to measure and how the relevant CATs can be quantified from metagenomic data.

## Conflict of Interest Statement

The authors declare that the research was conducted in the absence of any commercial or financial relationships that could be construed as a potential conflict of interest.
